# Short-term adverse events following modified vaccinia Ankara–Bavarian Nordic (MVA-BN) mpox vaccine with and without concomitant vaccines, Australia, 2022 to 2023

**DOI:** 10.2807/1560-7917.ES.2026.31.44.2600181

**Published:** 2026-11-05

**Authors:** Thuy Nguyen, Kristine Macartney, Nicholas Wood, Lucy Dawes, Lucy Deng

**Affiliations:** 1 National Centre for Immunisation Research and Surveillance, Westmead, New South Wales, Australia; 2 The University of Sydney Children's Hospital Westmead Clinical School, Westmead, New South Wales, Australia

**Keywords:** Mpox, Concomitant vaccination, Vaccine safety, Active surveillance, Australia

## Abstract

**BACKGROUND:**

Modified vaccinia Ankara–Bavarian Nordic (MVA-BN) mpox vaccine is used globally for disease prevention. Real-world safety data following co-administration of MVA-BN with other vaccines is limited.

**AIM:**

To characterise the adverse event profile of MVA-BN given alone or concomitantly with other vaccines using data from Australia’s active vaccine safety surveillance (AusVaxSafety).

**METHODS:**

Individuals (≥ 18 years) who received MVA-BN between 30 August 2022 and 20 April 2023 at sentinel AusVaxSafety surveillance sites and registered via QR code were surveyed on days 7 and 14 post-vaccination regarding adverse events following immunisation (AEFI), medical review by a healthcare professional and impact on daily activities. Analyses included participants who completed both surveys. Outcomes were compared by administration route, dose and concomitant vaccination status.

**RESULTS:**

Among 10,943 participants (response rate: 46%; 97% male), 200 (1.8%) received at least one concomitant vaccine. Across 14 days, AEFI rates were comparable or lower in concomitant recipients following intradermal administration (alone: 56% (Dose 1), 36% (Dose 2) vs concomitant: 53% (Dose 1), 32% (Dose 2)) and subcutaneous administration (alone: 49% (Dose 1), 32% (Dose 2) vs concomitant: 28% (Dose 1) and 24% (Dose 2)). Medical review was uncommon (0–2.7%). Impact on daily activities was low across groups (6% vs 3.5%), with most symptoms lasting ≤ 1 day.

**CONCLUSION:**

The frequency and severity of AEFIs within 14 days post-vaccination were similar between concomitant and non-concomitant group. These findings provide preliminary, exploratory evidence supporting the safe co-administration of MVA-BN with other adult vaccines, primarily in adult men, and may help inform future immunisation policy and public health recommendations.

**Key public health message**

**What did you want to address in this study and why?**

As mpox vaccines are increasingly used globally for prevention beyond outbreak responses, understanding the safety of co-administration is essential to inform mpox vaccination policies, as evidence is limited. This study aimed to assess the safety of the MVA-BN mpox vaccine when given alone or together with other vaccines in adults in Australia between August 2022 and April 2023.

**What have we learnt from this study?**

In over 10,000 people studied (97% men; 1.8% received MVA-BN with other vaccines), 45.7% reported adverse events following MVA-BN vaccination; most were generally mild and short-lived. Rates of adverse events, medical consultations and impact on daily activities were similar when MVA-BN was given together with other vaccines or alone. This was consistent across dose and administration route. Most impacts on daily activities such as missing work, lasted 1 day or less.

**What are the implications of your findings for public health?**

These findings provide preliminary, exploratory evidence supporting the safe co-administration of MVA-BN with other vaccines in adult men. However, the small proportion of participants receiving co-administered vaccines warrants cautious interpretation. The findings may offer some reassurance for both healthcare providers and recipients. They also form evidence to guide global immunisation policy and public health recommendations.

## Introduction

Mpox re-emerged globally in 2022 with ca 180,000 confirmed cases by 30 April 2022 [1]. In Australia, the first mpox outbreak occurred in 2022 with 144 cases reported nationally [2]. A larger resurgence was observed in 2024, with more than 1,400 cases recorded, and most reported infections were acquired locally [2]. In response, Australia implemented targeted vaccination strategies for populations at increased risk of exposure. Modified vaccinia Ankara–Bavarian Nordic (MVA-BN) mpox vaccine is being used in Australia for both primary preventive and post-exposure vaccination against mpox. 

The recommended primary course since 24 August 2022 in Australia consists of two subcutaneous (SC) doses of 0.5 mL or two intradermal (ID) doses of 0.1 mL as a dose-sparing alternative given 4 weeks apart [3]. Route of administration may influence reactogenicity because of differences in local immune response and antigen content delivery [4,5]. Accordingly, SC and ID administration of the MVA-BN vaccine are considered two distinct vaccination strategies. Different countries have implemented either SC or ID schedules depending on vaccine supply, with intradermal administration primarily used during periods of limited availability [6].

Evidence from clinical trials shows that MVA-BN is well tolerated across populations, including people with HIV or other immunocompromising conditions [7]. Commonly reported adverse events included local and systemic reactions of mild to moderate intensity that did not require additional treatment [8,9]. Early post-marketing surveillance from Australia, Germany and the United States has supported these findings, with most reported adverse events being mild and self-limiting [10-12]. However, real-world safety data following co-administration of MVA-BN with other vaccines, such as COVID-19, influenza, or routine adult vaccines, is limited. In the context of more widespread and ongoing use of mpox vaccines globally for disease prevention beyond outbreak responses in recent years, evidence on concomitant administration is vital for global vaccine safety groups to inform mpox vaccination policies.

To address current evidence gaps, we analysed AusVaxSafety data, Australia’s active vaccine safety surveillance system, to characterise the adverse event profile of MVA-BN given alone or concomitantly with other vaccines, stratified by SC and ID administration to account for differences in vaccination strategy and potential effects on reactogenicity.

## Methods

### Study population

Individuals aged 18 years and older who received MVA-BN between 30 August 2022 and 20 April 2023 at sentinel AusVaxSafety surveillance sites were invited to participate via QR code self-registration in our vaccine safety survey, as previously described [13]. Participants could contribute survey data following either Dose 1, Dose 2 or both doses. Surveillance sites accounted for 76% (43/57) of all mpox vaccination sites in Australia.

### Survey

Registered participants received survey links via SMS or email on day 7 following an MVA-BN vaccination encounter, regardless of dose number in their vaccination course. The survey asked participant to report adverse events following immunisation (AEFI), including predefined solicited symptoms and other symptoms (free text), any medical review (phone advice line, general practitioner or Aboriginal and Torres Strait Islander Health Worker or sexual health clinic, emergency department), or impact on daily activities (‘Did any of the symptoms you reported cause you to miss work, study or normal daily activities?’) occurring 0–7 days post-vaccination. Participants were also asked about underlying medical conditions (Day 7 survey questionnaire is provided in Supplementary Table S2). Participants who responded to the Day 7 survey were sent a follow-up survey on day 14 to report any AEFI, solicited symptoms, medical review, and impact on daily activities occurring 8–14 days post-vaccination (Day 14 survey questionnaire is provided in Supplementary Table S3). A reminder SMS or email was sent on days 8 and 15 if surveys were not completed. Responses were included for analysis if participants completed both Day 7 and Day 14 surveys within 7 days of survey distribution. Participant inclusion and exclusion are shown in Figure 1.

**Figure 1:**
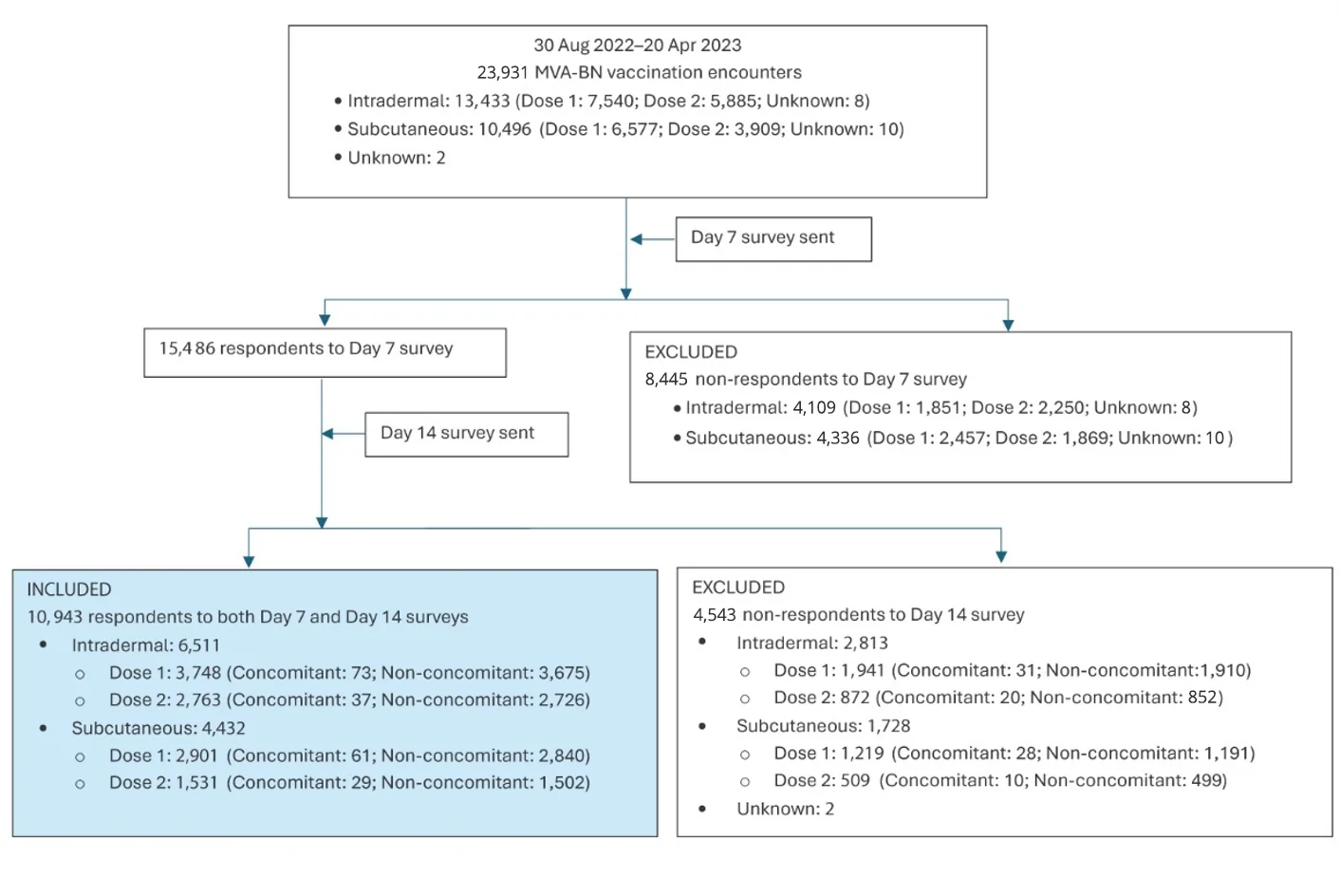
Flow diagram for inclusion and exclusion of study participants, Australia, 30 August 2022–20 April 2023 (n = 10,943)

MVA-BN: modified vaccinia Ankara–Bavarian Nordic mpox vaccine.

Concomitantly administered vaccines included COVID-19, hepatitis A and B, human papillomavirus (HPV), tetanus, typhoid, pneumococcal, meningococcal, influenza, and other travel vaccines (including but not limited to yellow fever and rabies).

### Variable

Vaccine brand (JYNNEOS, Bavarian Nordic), dose and date, site of vaccine administration, concomitant vaccines and demographic details including age and gender (self-identified as male, female or ‘another term’) in the survey were self-reported at registration. The survey enrolment form is provided in Supplementary Table S1. Concomitant administration was defined as one or more vaccines given by the same clinic/vaccination provider on the same day with MVA-BN mpox vaccine. Vaccinations were classified by reported site of administration and assumed to be ID if administered in the forearm and SC if administered in the deltoid.

### Data analysis

We analysed the proportion of respondents reporting AEFI, seeking medical review and missing daily activities after vaccination. Outcomes were summarised by route of administration, dose and concomitant vaccination status. Formal statistical comparisons between groups were not performed because of the small number of participants receiving concomitant vaccination and the heterogeneity of concomitant vaccine combinations. Findings were therefore summarised descriptively and should be interpreted as exploratory.

Descriptive analyses were conducted in R version 4.4.1 (R Foundation).

## Results

A total of 23,931 individuals aged 18 years and older who received an MVA-BN vaccine registered for surveillance, including 13,433 who received ID administration, and 10,496 who received SC administration (Figure 1). Among ID recipients, 6,511 (48%) respondents completed both the Day 7 and Day 14 surveys, compared with 4,432 (42%) among SC recipients. Only 200 respondents (1.8%) reported receiving at least one other vaccine during the same vaccination encounter as MVA-BN, most commonly COVID-19 (n = 38; 19%), hepatitis A (28; 14%), hepatitis B (28; 14%), human papilloma virus (HPV) (18; 9%), and combined hepatitis A and B vaccines (11; 6%). Of those receiving concomitant vaccines, most received one additional vaccine (n = 172; 86%), followed by two (25; 12.5%) or three or more additional vaccines (3; 1.5%). The median age was 42 (interquartile range (IQR): 33–54) years, 97% identified as male. Additional demographic details are presented in Supplementary Table S4.

### Adverse events

Overall, the AEFI rates were similar or lower among concomitant vaccine recipients compared with those receiving MVA-BN alone, regardless of administration route or dose.

Following MVA-BN ID vaccination, the day 0–7 reported AEFI rate after Dose 1 was 53% when given alone vs 48% when given concomitantly (Table 1), decreasing to 33% for day 8–14 for both. After Dose 2, the day 0–7 AEFI rates were 34% (alone) vs 30% (concomitant), decreasing to 13% and 8.1% respectively for day 8–14 (Table 1). Across the 14-day surveillance period, 2–3% of participants reported new AEFI between day 8–14, while 7–30% reported having ongoing symptoms beyond day 7 (Figure 2).

**Table 1:** Adverse events following 0.1-ml intradermal MVA-BN vaccination given with or without concomitant vaccination^a^, Australia, 30 August 2020–20 April 2023 (n = 6,511)

Outcome	MVA-BN 0.1-mL intradermal given alone (n = 6,401)														MVA-BN 0.1-mL intradermal given concomitantly (n = 110)													
	Dose 1 (n = 3,675)							Dose 2^b^ (n = 2,726)							Dose 1 (n = 73)							Dose 2^b^ (n = 37)						
	N^c^	D0–7^d^		D8–14^e^		D0–14^f^		N^c^	D0–7^d^		D8–14^e^		D0–14^f^		N^c^	D0–7^d^		D8–14^e^		D0–14^f^		N^c^	D0–7^d^		D8–14^e^		D0–14^f^	
		n	%	n	%	n	%		n	%	n	%	n	%		n	%	n	%	n	%		n	%	n	%	n	%
**Any adverse event**	3,675	1,951	53	1,222	33	2,075	56	2,726	937	34	359	13	979	36	73	35	48	24	33	39	53	37	11	30	3	8.1	12	32
**Age (years) **																												
18–39	1,735	964	56	629	36	1,025	59	1,074	391	36	153	14	405	38	43	22	51	17	40	23	53	25	8	32	2	8.0	9	36
40–59	1,471	796	54	499	34	847	58	1,226	426	35	169	14	448	37	26	12	46	7	27	15	58	9	2	22	1	11	2	22
≥ 60	469	191	41	94	20	203	43	426	120	28	37	8.7	126	30	4	1	25	0	0	1	25	3	1	33	0	0	1	33
**Gender^g^**																												
Male	3,571	1,884	53	1,183	33	2,003	56	2,674	918	34	350	13	959	36	72	35	49	24	33	39	54	37	11	30	3	8.1	12	32
Female	72	49	68	30	42	52	72	37	13	35	4	11	13	35	1	0	0	0	0	0	0	0	0	NA	0	NA	0	NA
Another term	32	18	56	9	28	20	63	15	6	40	5	33	7	47	0	0	NA	0	NA	0	NA	0	0	NA	0	NA	0	NA
**Any chronic medical condition^h^**																												
Yes	562	329	59	197	35	351	62	437	185	42	55	13	189	43	8	6	75	2	25	6	5	6	4	67	1	17	4	67
No	3,113	1,622	52	1,025	33	1,724	55	2,289	752	33	304	13	790	35	65	29	45	22	34	33	51	31	7	23	2	6.5	8	26
**Medical review^i^**	3,675	34	0.9	26	0.7	51	1.4	2,726	24	0.9	9	0.3	28	1.0	73	1	1.4	0	0	1	1.4	37	1	2.7	0	0	1	2.7
**Highest level of medical advice sought^j^**																												
Phone	NA	3	8.8	5	19	4	7.8	NA	5	21	2	22	3	11	NA	0	0	NA	NA	0	0	NA	0	0	NA	NA	0	0
GP/Aboriginal HCW/SHC	NA	26	76	17	65	31	61	NA	16	67	5	56	16	57	NA	1	100	NA	NA	1	100	NA	1	100	NA	NA	1	100
ED	NA	5	15	4	15	7	14	NA	3	13	2	22	4	14	NA	0	0	NA	NA	0	0	NA	0	0	NA	NA	0	0
Reported on both survey^k^	NA	NA	NA	NA	NA	9	18	NA	NA	NA	NA	NA	5	18	NA	NA	NA	NA	NA	0	0	NA	NA	NA	NA	NA	0	0
**Impact on daily activities**	3,675	112	3.0	35	1.0	136	3.7	2,726	62	2.3	21	0.8	69	2.5	73	4	5.5	1	1.4	4	5.5	37	2	5.4	2	5.4	2	5.4
**Days impacted^l^**																												
1 day or less	NA	59	53	14	40	69	51	NA	39	63	8	38	39	57	NA	1	25	0	0	1	25	NA	1	50	1	50	0	0
2 days or more	NA	53	47	21	60	67	49	NA	23	37	13	62	30	43	NA	3	75	1	100	3	75	NA	1	50	1	50	2	100

ED: emergency department; HCW: healthcare worker; GP: general practitioner; MVA-BN: modified vaccinia Ankara–Bavarian Nordic mpox vaccine; SHC: sexual health centre.

^a^ Concomitantly administered vaccines included COVID-19, hepatitis A and B, human papilloma virus (HPV), tetanus, typhoid, pneumococcal, meningococcal, influenza, and other travel vaccines (including but not limited to yellow fever and rabies).

^b^ Dose 2 was given 28 days after Dose 1.

^c^ Denominators for adverse events following immunisation (AEFI) rate calculations, representing the number of respondents within each subgroup.

^d^ D0–7: Number of respondents reporting a reaction within 7 days after vaccination, based on the Day 7 survey.

^e^ D8–14: Number of respondents reporting a reaction between days 8 and 14 after vaccination, based on the Day 14 survey.

^f^ D0–14: Number of respondents reporting any reaction within 14 days after vaccination, combining data from the Day 7 and Day 14 surveys.

^g^ Gender refers to a person's self-identified gender rather than their biological sex.

^h^ Chronic medical condition was assessed using a Yes/No question, with respondents able to select from a list of conditions provided in Supplementary Table S2.

^i^ Medical review: respondent answered 'Yes' to the question 'Did you need to seek medical care/advice for any of your symptoms?' and ticked 'Phone advice', 'Care from a GP, Aboriginal Healthcare Worker or sexual health clinic' or 'Visit to a hospital emergency department'.

^j^ Denominator is restricted to those who reported any medical reviews.

^k^ Following non-concomitant Dose 1, all nine respondents used the same service type in both surveys (phone: n = 2; GP/Aboriginal HCW/SHC:  n = 6; ED: n = 1); following non-concomitant Dose 2, most used the same service type (phone, n = 2, GP/Aboriginal HCW/SHC, n = 2), while one respondent sought GP first and ED in the second survey.

^l^ Denominator is restricted to those who reported any impacts on daily activities.

**Figure 2:**
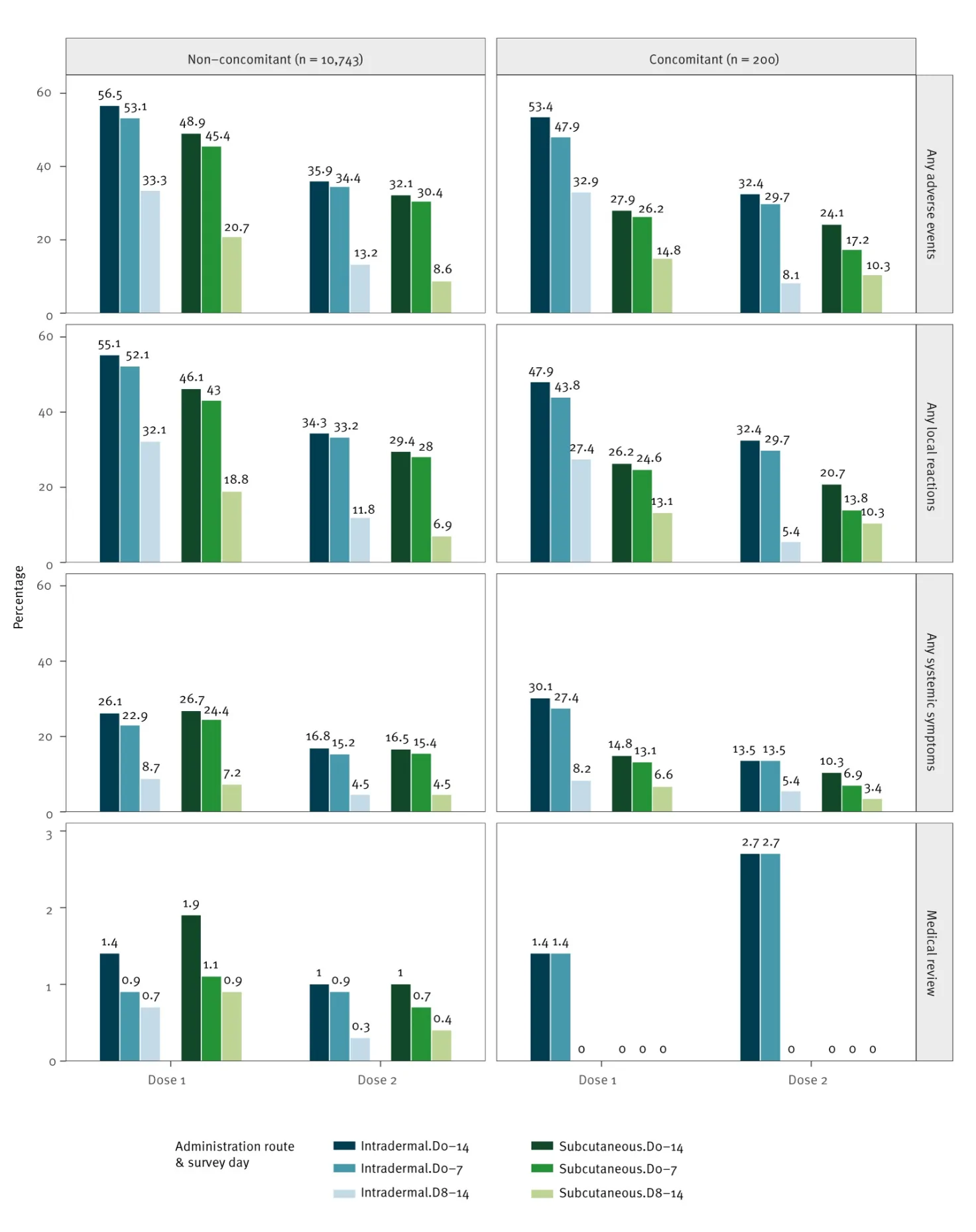
Proportion of respondents reporting adverse events following MVA-BN vaccine by concomitant vaccination status, administration route, vaccine dose and survey time point, Australia, 30 August 2020–20 April 2023 (n = 10,943)

MVA-BN: modified vaccinia Ankara–Bavarian Nordic mpox vaccine.

Concomitantly administered vaccines included COVID-19, hepatitis A and B, human papillomavirus (HPV), tetanus, typhoid, pneumococcal, meningococcal, influenza, and other travel vaccines (including but not limited to yellow fever and rabies)

Following MVA-BN SC vaccination, the day 0–7 AEFI rate after Dose 1 was 45% (alone) vs 26% (concomitant), decreasing to 21% and 15% respectively for day 8–14 (Table 2). After Dose 2, AEFI rates were 30% vs 17% for day 0–7, and 8.6% vs 10% for day 8–14 (Table 2). Across the 14-day surveillance period, 1–5% of participants reported new AEFI between day 8–14, while 7–15% reported having ongoing symptoms beyond day 7 (Figure 2).

**Table 2:** Adverse events following 0.5-mL subcutaneous MVA-BN vaccination given with or without concomitant vaccination^a^, Australia, 30 August 2020–20 April 2023 (n = 4,432)

Outcome	MVA-BN 0.5-mL subcutaneous given alone (n = 4,342)														MVA-BN 0.5-mL subcutaneous given concomitantly (n = 90)													
	Dose 1 (n = 2,840)							Dose 2^b^ (n = 1,502)							Dose 1 (n = 61)							Dose 2^b^ (n = 29)						
	N^c^	D0–7^d^		D8–14^e^		D0–14^f^		N^c^	D0–7^d^		D8–14^e^		D0–14^f^		N^c^	D0–7^d^		D8–14^e^		D0–14^f^		N^c^	D0–7^d^		D8–14^e^		D0–14^f^	
		n	%	n	%	n	%		n	%	n	%	n	%		n	%	n	%	n	%		n	%	n	%	n	%
**Any adverse event**	2,840	1,289	45	589	21	1,390	49	1,502	456	30	129	8.6	482	32	61	16	26	9	15	17	28	29	5	17	3	10	7	24
**Age (years) **																												
18–39	1,194	574	48	260	22	618	52	575	204	35	54	9.4	212	37	30	8	27	4	13	8	27	14	3	21	1	7.1	3	21
40–59	1,254	574	46	269	21	617	49	703	204	29	61	8.7	219	31	25	7	28	3	12	7	28	6	2	33	0	0	2	33
≥ 60	392	141	36	60	15	155	40	224	48	21	14	6.3	51	23	6	1	17	2	33	2	33	9	0	0	2	22	2	22
**Gender^g^**																												
Male	2,726	1,222	45	551	20	1,318	48	1,454	433	30	127	8.7	459	32	60	16	27	9	15	17	28	27	5	19	3	11	7	26
Female	87	56	64	32	37	59	68	42	21	50	2	4.8	21	50	1	0	0	0	0	0	0	2	0	0	0	0	0	0
Another term	27	11	41	6	22	13	48	6	2	33	0	0	2	33	0	NA	NA	NA	NA	NA	NA	0	NA	NA	NA	NA	NA	NA
**Any chronic medical condition^h^**																												
Yes	512	244	48	128	25	262	51	232	87	38	33	14	92	40	14	4	29	3	21	4	29	11	1	9.1	2	18	3	27
No	2,328	1,045	45	461	20	1,128	48	1,270	369	29	96	7.6	390	31	47	12	26	6	13	13	28	18	4	22	1	5.6	4	22
**Medical review^i^**	2,840	32	1.1	26	0.9	53	1.9	1,502	10	0.7	6	0.4	15	1.0	61	0	0	0	0	0	0	29	0	0	0	0	0	0
**Highest level of medical advice sought^j^**																												
Phone	NA	8	25	7	27	12	23	NA	1	10	0	0	1	6.7	NA	NA	NA	NA	NA	NA	NA	NA	NA	NA	NA	NA	NA	NA
GP/Aboriginal HCW/SHC	NA	24	75	17	65	34	64	NA	7	70	6	100	11	73	NA	NA	NA	NA	NA	NA	NA	NA	NA	NA	NA	NA	NA	NA
ED	NA	0	0	2	7.7	2	3.8	NA	2	20	0	0	2	13	NA	NA	NA	NA	NA	NA	NA	NA	NA	NA	NA	NA	NA	NA
Reported on both survey^k^	NA	NA	NA	NA	NA	5	9	NA	NA	NA	NA	NA	1	6.7	NA	NA	NA	NA	NA	NA	NA	NA	NA	NA	NA	NA	NA	NA
**Impact on daily activities**	2,840	109	3.8	29	1.0	125	4.4	NA	42	2.8	14	0.9	48	3.2	61	1	1.6	1	1.6	1	1.6	29	1	3.4	0	0	1	3.4
**Days impacted^l^**																												
1 day or less	NA	69	63	11	38	71	57	NA	30	71	6	43	29	60	NA	0	0	1	100	0	0	NA	1	100	NA	NA	1	100
2 days or more	NA	40	37	18	62	54	43	NA	12	29	8	57	19	40	NA	1	100	0	0	1	100	NA	0	0	NA	NA	0	0

ED: emergency department; HCW: healthcare worker; GP: general practitioner; MVA-BN: modified vaccinia Ankara–Bavarian Nordic mpox vaccine; SHC: sexual health centre.

^a^ Concomitantly administered vaccines included COVID-19, hepatitis A and B, human papilloma virus (HPV), tetanus, typhoid, pneumococcal, meningococcal, influenza, and other travel vaccines (including but not limited to yellow fever and rabies).

^b^ Dose 2 was given 28 days after Dose 1.

^c^ Denominators for adverse events following immunisation (AEFI) rate calculations, representing the number of respondents within each subgroup.

^d^ D0–7: Number of respondents reporting a reaction within 7 days after vaccination, based on the Day 7 survey.

^e^ D8–14: Number of respondents reporting a reaction between Days 8 and 14 after vaccination, based on the Day 14 survey.

^f^ D0–14: Number of respondents reporting any reaction within 14 days after vaccination, combining data from the Day 7 and Day 14 surveys.

^g^ Gender refers to a person's self-identified gender rather than their biological sex.

^h^ Chronic medical condition was assessed using a Yes/No question, with respondents able to select from a list of conditions provided in Supplementary Table S2.

^i^ Medical review: respondent answered 'Yes' to the question 'Did you need to seek medical care/advice for any of your symptoms?' and ticked 'Phone advice', 'Care from a GP, Aboriginal Healthcare Worker or sexual health clinic' or 'Visit to a hospital emergency department'.

^j^ Denominator is restricted to those who reported any medical reviews.

^k^ Following non-concomitant Dose 1, all nine respondents used the same service type in both surveys (phone: n = 2; GP/Aboriginal HCW/SHC:  n = 6; ED: n = 1); following non-concomitant Dose 2, most used the same service type (phone, n = 2, GP/Aboriginal HCW/SHC, n = 2), while one respondent sought GP first and ED in the second survey.

^l^ Denominator is restricted to those who reported any impacts on daily activities.

### Solicited symptoms

Local reactions were the most frequently reported AEFIs following both IC (alone: 34–55%, concomitant: 32–48%; Table 3) and SC administration (alone: 29–46%, concomitant: 21–26%; Table 4) across doses over the 14-day period. Fatigue was the most common systemic symptom over the 14-day period across both ID (6.9–23%), while cardiorespiratory symptoms such as chest symptoms, shortness of breath or palpitation were rare (< 2%). Local symptoms (local pain, itching, redness, and swelling) were the most common persistent symptoms beyond day 7, with 10–30% of participants reporting ongoing symptoms up to 14 days post-vaccination, regardless of concomitant vaccine receipt.

**Table 3:** Solicited adverse event rates following 0.1-mL intradermal MVA-BN vaccination given with or without concomitant vaccination^a^, Australia, 30 August 2020–20 April 2023 (n = 6,511)

Symptoms	MVA-BN 0.1-mL intradermal given alone (n = 6,401)												MVA-BN 0.1-mL intradermal given concomitantly (n = 110)											
	Dose 1 (n = 3,675)						Dose 2^b^ (n = 2,726)						Dose 1 (n = 73)						Dose 2^b^ (n = 37)					
	D0–7^c^		D8–14^d^		D0–14^e^		D0–7^c^		D8–14^d^		D0–14^e^		D0–7^b^		D8–14^d^		D0–14^e^		D0–7^c^		D8–14^d^		D0–14^e^	
	n	%	n	%	n	%	n	%	n	%	n	%	n	%	n	%	n	%	n	%	n	%	n	%
Local all	1,914	52	1,180	32	2,026	55	905	33	321	12	935	34	32	44	20	27	35	48	11	30	2	5.4	12	32
Local pain	494	13	133	3.6	525	14	168	6.2	25	0.9	178	6.5	7	9.6	1	1.4	7	9.6	3	8.1	0	0	3	8.1
Local itching	1,462	40	569	15	1,561	42	696	26	152	5.6	726	27	26	36	13	18	29	40	5	14	1	2.7	6	16
Local redness	1,812	49	1,057	29	1,922	52	791	29	273	10	819	30	30	41	17	23	33	45	9	24	1	2.7	10	27
Local swelling	1,408	38	732	20	1,508	41	613	22	130	4.8	641	24	22	30	14	19	26	36	8	22	2	5.4	9	24
Lymphadenopathy^f^	67	1.8	37	1.0	83	2.3	20	0.7	7	0.3	24	0.9	0	0	1	1.4	1	1.4	0	0	0	0	0	0
Systemic all	843	23	318	8.7	960	26	414	15	122	4.5	457	17	20	27	6	8.2	22	30	5	14	2	5.4	5	14
Myalgia/arthralgia	296	8.1	103	2.8	349	9.5	158	5.8	43	1.6	175	6.4	4	5.5	1	1.4	5	6.8	2	5.4	0	0	2	5.4
Headache	321	8.7	96	2.6	374	10	165	6.1	41	1.5	180	6.6	6	8.2	3	4.1	8	11	1	2.7	0	0	1	2.7
Fever^g^	94	2.6	27	0.7	113	3.1	55	2.0	15	0.6	61	2.2	2	2.7	0	0	2	2.7	1	2.7	0	0	1	2.7
Chills	93	2.5	31	0.8	113	3.1	51	1.9	11	0.4	57	2.1	2	2.7	0	0	2	2.7	0	0	0	0	0	0
Fatigue	615	17	205	5.6	696	19	320	12	80	2.9	343	13	16	22	5	6.8	17	23	5	14	2	5.4	5	14
Gastrointestinal^h^	156	4.2	74	2.0	197	5.4	79	2.9	16	0.6	87	3.2	3	4.1	3	4.1	5	6.8	2	5.4	1	2.7	2	5.4
Chest^i^	47	1.3	12	0.3	55	1.5	16	0.6	8	0.3	22	0.8	0	0	0	0	0	0	2	5.4	0	0	2	5.4
Shortness of breath	49	1.3	23	0.6	65	1.8	29	1.1	13	0.5	35	1.3	1	1.4	1	1.4	2	2.7	0	0	0	0	0	0
Palpitations	35	1.0	9	0.2	41	1.1	10	0.4	5	0.2	12	0.4	0	0	0	0	0	0	0	0	0	0	0	0
Other^j^	112	3.0	70	1.9	165	4.5	50	1.8	26	1.0	72	2.6	5	6.8	1	1.4	6	8.2	0	0	0	0	0	0

MVA-BN: modified vaccinia Ankara–Bavarian Nordic mpox vaccine.

^a^ Concomitantly administered vaccines included COVID-19, hepatitis A and B, Human papilloma virus (HPV), tetanus, typhoid, pneumococcal, meningococcal, influenza, and other travel vaccines (including but not limited to yellow fever and rabies).

^b^ Dose 2 was given 28 days after Dose 1.

^c^ D0–7: Number of respondents reporting a reaction within 7 days after vaccination, based on the Day 7 survey.

^d^ D8–14: Number of respondents reporting a reaction between days 8 and 14 after vaccination, based on the Day 14 survey.

^e^ D0–14: Number of respondents reporting any reaction within 14 days after vaccination, combining data from the Day 7 and Day 14 surveys.

^f^ Lymphadenopathy was defined as swelling in the armpit area.

^g^ Fever was defined as a temperature > 38 °C.

^h^ Gastrointestinal symptoms include nausea, vomiting, diarrhoea, and abdominal pain.

^i^ Chest symptoms include chest pain, pressure, or discomfort.

^j^ Unsolicited (other) events including injection site mass, nasopharyngitis and pruritus, among other symptoms self-reported in free text.

**Table 4:** Solicited adverse event rates following 0.5-mL subcutaneous MVA-BN vaccination given with or without concomitant vaccination^a^, Australia, 30 August 2020–20 April 2023 (n = 4,432)

Symptoms	MVA-BN 0.5-mL subcutaneous given alone (n = 4,342)												MVA-BN 0.5-mL subcutaneous given concomitantly (n = 90)											
	Dose 1 (n = 2,840)						Dose 2^b^ (n = 1,502)						Dose 1 (n = 61)						Dose 2^b^ (n = 29)					
	D0–7^c^		D8–14^d^		D0–14^e^		D0–7^c^		D8–14^d^		D0–14^e^		D0–7^b^		D8–14^d^		D0–14^e^		D0–7^c^		D8–14^d^		D0–14^e^	
	n	%	n	%	n	%	n	%	n	%	n	%	n	%	n	%	n	%	n	%	n	%	n	%
Local all	1,220	43	535	19	1,309	46	421	28	103	6.9	441	29	15	25	8	13	16	26	4	14	3	10	6	21
Local pain	870	31	237	8.3	918	32	222	15	46	3.1	237	16	10	16	1	1.6	11	18	2	6.9	2	6.9	3	10
Local itching	465	16	167	5.9	499	18	220	15	45	3.0	234	16	7	11	3	4.9	8	13	2	6.9	2	6.9	4	14
Local redness	644	23	229	8.1	693	24	272	18	65	4.3	284	19	11	18	4	6.6	11	18	2	6.9	2	6.9	4	14
Local swelling	750	26	384	14	841	30	262	17	58	3.9	276	18	10	16	5	8.2	10	16	3	10	0	0	3	10
Lymphadenopathy^f^	53	1.9	16	0.6	58	2.0	20	1.3	7	0.5	23	1.5	1	1.6	0	0	1	1.6	0	0	0	0	0	0
Systemic all	694	24	204	7.2	757	27	231	15	67	4.5	248	17	8	13	4	6.6	9	15	2	6.9	1	3.4	3	10
Myalgia/arthralgia	278	9.8	77	2.7	312	11	98	6.5	39	2.6	112	7.5	4	6.6	3	4.9	5	8.2	2	6.9	0	0	2	6.9
Headache	271	9.5	60	2.1	299	11	94	6.3	28	1.9	101	6.7	4	6.6	2	3.3	4	6.6	1	3.4	1	3.4	2	6.9
Fever^g^	77	2.7	12	0.4	81	2.9	21	1.4	6	0.4	23	1.5	1	1.6	0	0	1	1.6	0	0	0	0	0	0
Chills	75	2.6	16	0.6	84	3.0	24	1.6	8	0.5	29	1.9	2	3.3	0	0	2	3.3	0	0	0	0	0	0
Fatigue	498	18	127	4.5	540	19	172	11	48	3.2	185	12	6	9.8	1	1.6	6	9.8	1	3.4	1	3.4	2	6.9
Gastrointestinal^h^	108	3.8	37	1.3	127	4.5	44	2.9	14	0.9	50	3.3	2	3.3	1	1.6	3	4.9	0	0	0	0	0	0
Chest^i^	26	0.9	23	0.8	42	1.5	7	0.5	6	0.4	12	0.8	0	0	0	0	0	0	0	0	0	0	0	0
Shortness of breath	33	1.2	19	0.7	43	1.5	9	0.6	8	0.5	13	0.9	1	1.6	0	0	1	1.6	0	0	0	0	0	0
Palpitations	19	0.7	12	0.4	26	0.9	5	0.3	3	0.2	6	0.4	0	0	1	1.6	1	1.6	0	0	0	0	0	0
Other^j^	116	4.1	62	2.2	159	5.6	23	1.5	11	0.7	30	2.0	1	1.6	0	0	1	1.6	0	0	0	0	0	0

MVA-BN: modified vaccinia Ankara–Bavarian Nordic mpox vaccine.

^a^ Concomitantly administered vaccines included COVID-19, hepatitis A and B, Human papilloma virus (HPV), tetanus, typhoid, pneumococcal, meningococcal, influenza, and other travel vaccines (including but not limited to yellow fever and rabies).

^b^ Dose 2 was given 28 days apart from Dose 1.

^c^ D0–7: Number of respondents reporting a reaction within 7 days after vaccination, based on the Day 7 survey.

^d^ D8–14: Number of respondents reporting a reaction between days 8 and 14 after vaccination, based on the Day 14 survey.

^e^ D0–14: Number of respondents reporting any reaction within 14 days after vaccination, combining data from the Day 7 and Day 14 surveys.

^f^ Lymphadenopathy was defined as swelling in the armpit area.

^g^ Fever was defined as a temperature > 38 °C.

^h^ Gastrointestinal symptoms include nausea, vomiting, diarrhoea, and abdominal pain.

^i^ Chest symptoms include chest pain, pressure, or discomfort.

^j^ Unsolicited (other) events including injection site mass, nasopharyngitis, and pruritus, among other symptoms self-reported in free text.

Across all survey time points, doses and concomitant vaccination groups, the point estimates of any AEFI and solicited symptom rates were consistently higher following ID compared with SC administration. The values of AEFI rates declined following the second dose compared with the first, irrespective of vaccination route or concomitant vaccine receipt. Within the non-concomitant group, older participants, males, and participants with no chronic medical conditions reported slightly fewer AEFIs than their comparison groups, regardless of vaccination route, survey time point, or dose. Only a limited number of subgroup analyses within the concomitant group were conducted due to the small number of participants in each subgroup.

### Medical review

Medical review rates were rare across all subgroups. Among concomitant recipients, medical review was reported at day 7 by ID recipients (Dose 1: 1.4%; Dose 2: 2.7%) but not by SC recipients, and no ID or SC concomitant recipients reported medical review at day 14 following either dose. Among non-concomitant group, medical review rates were 0.9% for both Dose 1 and Dose 2 at day 0–7, and 0.7% (Dose 1) and 0.3% (Dose 2) at day 8–14 following ID administration (Table 1). For SC recipients, the rates were 1.1% (Dose 1) and 0.7% (Dose 2) at day 7 and 0.9% (Dose 1) and 0.4% at (Dose 2) at day 14 (Table 2). Only a small number of participants (n = 20) reported ongoing symptoms requiring repeated medical review following either ID or SC administration.

Concomitant vaccine recipients reported similar rates of impact on daily activities over the 14-day period (ID: 3.7–5.5%, SC: 3.2–4.4%) compared with non-concomitant participants (ID: 0.8–5.4%, SC: 1.6–3.4%), and more than half of respondents across all groups indicated that any impact on daily activities lasted for one day or less.

## Discussion

This post-marketing study investigates adverse events following MVA-BN vaccination, and, to our knowledge, is the first to assess concomitant administration. We found that the frequency and severity of AEFIs within 14 days post-vaccination were similar among those received MVA-BN alone and those received MVA-BN concomitantly with other vaccines. Although only a small proportion of participants reported receiving concomitant vaccines, the values of their self-reported AEFI rates were similar to those receiving MVA-BN alone. Medical review and impacts on daily activities were rare in both groups, providing preliminary, exploratory evidence on the safe co-administration of MVA-BN with other vaccines, primarily in adult men. These findings are particularly important given that some people eligible for mpox vaccine may experience barriers to accessing healthcare [3]. The ability to receive multiple vaccines in a single visit could therefore improve uptake and individual protection against a range of important diseases [14]. Moreover, for individuals receiving MVA-BN as pre-exposure prophylaxis before travel, concomitant vaccination allows for broader protection against diverse potential exposures in a timely way.

Overall, AEFI patterns in both concomitant and non-concomitant groups aligned with MVA-BN reactogenicity profiles reported in clinical trials and other observational data. Higher rates were observed following ID compared with SC administration, and AEFI reporting decreased after the second dose [10-12]. Notably, ID administration is now used less frequently as initial supply constraints have been resolved [15]. Local reactions were the most frequently reported AEFIs, systemic symptoms were less common, and cardiorespiratory symptoms remained rare (< 2%), in line with prior evidence [8-11,16,17]. Impact on daily activities was infrequent and lower than that reported for other routine adult vaccines monitored in Australia, although the observation period for routine vaccines was shorter (0–3 days post-vaccination) [13,18,19].

By linking participant responses at day 7 and day 14, this study was able to capture both early (0–7 days) and delayed (8–14 days) adverse events, as well as the proportion of participants experiencing sustained reactions across the 14-day follow-up. The results indicate that most adverse events occurred within the first 7 days, with few delayed events reported between day 8 and day 14, and only a small proportion of participants in both concomitant and non-concomitant groups experienced symptoms persisting across the two-week period. These findings demonstrate that adverse events were generally short-lived and that the frequency and duration of sustained reactions were similar following co-administration. Importantly, although myocarditis has been previously reported following MVA-BN vaccine [20], no cases were identified in either the MVA-BN given alone or concomitantly groups in our study.

This study has several limitations. Firstly, only a small proportion of participants reported receiving concomitant vaccination (n = 200; 1.8%), limiting statistical power and generalisability. The observed differences in AFI rates may reflect chance variation because of the small sample size, residual confounding or heterogeneity in concomitant vaccine combinations, and should therefore be interpreted as descriptive and exploratory. In addition, the small numbers of specific vaccine combinations, mainly COVID-19 vaccines and hepatitis vaccines, precluded meaningful comparisons by individual vaccine type. Therefore, no conclusions can be made on the safety profile of specific concomitant combinations. Secondly, limitation of this design is that participants who did not complete both surveys were excluded, which may introduce bias if non-respondents systematically differed in their experience of adverse events. However, the demographic characteristics of excluded participants, provided in Supplementary Table S5, were similar to those included in the analysis. Participation was also restricted to individuals who were fluent in English and had internet access. In addition, participants who chose to receive concomitant vaccination may have differed from those who received MVA-BN alone in ways that were not measured, including their health-seeking behaviour, vaccination preferences or tolerance of adverse events. If individuals receiving concomitant vaccines were less likely to perceive or report mild symptoms, AEFI rates following concomitant vaccination may have been underestimated. Thirdly, because vaccination encounters were anonymous, Dose 1 and Dose 2 could not be linked at the individual level and were therefore analysed as independent events. Rates of self-reported events should not be interpreted as being causally related to vaccine administration; while this may be more likely for injection site reactions, systemic and other symptoms cannot be confirmed as attributable to vaccination in our system, and no control group exists. Finally, AusVaxSafety collects de-identified, self-reported short-term solicited adverse events following immunisation and does not capture the individual-level validated clinical data required for formal causality assessment using the WHO algorithm. As such, conducting a formal causality assessment is not feasible within the scope of this active sentinel surveillance system and is outside the intended design of this study.

These observational data do not replace clinical studies evaluating concomitant vaccination. However, in the absence of such studies, they provide valuable real-world evidence to inform vaccine safety monitoring and policy decisions. Given the cost and complexity of conducting dedicated clinical studies in this area, such observational data remain an important source of evidence.

## Conclusion

Overall, the combination of a large sample size, active follow-up, and linked day 7/day 14 data provide evidence of both the safety and temporal profile of MVA-BN vaccination, including when administered concomitantly with other vaccines. These findings support the safe co-administration of MVA-BN with other adult vaccines and provide evidence to guide immunisation policy and public health recommendations.

## Data Availability

De-identified individual participant data underpinning the findings of this study may be made available upon request, pending approval from the data owner. Requests for data, accompanied by a methodologically sound proposal, can be submitted to AusVaxSafety. The study group will review all applications for data access. Upon approval, the data will be shared exclusively for the purposes outlined in the approved proposal. Data requesters must sign a data access agreement before gaining access.

## Supplementary Data

153000 Supplement
