# Supplementary material for: Short-term adverse events following modified vaccinia Ankara–Bavarian Nordic (MVA-BN) mpox vaccine with and without concomitant vaccines, Australia, 2022 to 2023
Source: Euro Surveill. 2026 Nov 5;31(44):2600181. doi: 10.2807/1560-7917.ES.2026.31.44.2600181 (PMC13743899; doi:10.2807/1560-7917.ES.2026.31.44.2600181)
Supplement: Supplementary file 1 [file 26-00181_NGUYEN_Supplement.pdf]

This supplementary material is hosted by *Eurosurveillance* as supporting information alongside the article *Short-term adverse events following modified vaccinia Ankara–Bavarian Nordic (MVA-BN) mpox vaccine with and without concomitant vaccines, Australia, 2022 to 2023*, on behalf of the authors, who remain responsible for the accuracy and appropriateness of the content. The same standards for ethics, copyright, attributions and permissions as for the article apply. Supplements are not edited by *Eurosurveillance* and the journal is not responsible for the maintenance of any links or email addresses provided therein.

Supplement Table S1. Survey enrolment form

Question	Response options
**Demographics**	
What is your gender?	M, Male<br>F, Female<br>O, Another term
Do you identify as Aboriginal or Torres Strait Islander?	1, Aboriginal<br>2, Torres Strait Islander<br>3, Both<br>0, Neither
Are you currently pregnant?	1. Yes<br>0. No<br>2. Not applicable
**Vaccine**	
What is the brand of the vaccine?	1. ACAM2000<br>2. Jynneos
Which vaccine dose was this?	1. First<br>2. Second
What was the site of vaccine administration?	1. Left arm subcutaneous<br>2. Left arm intradermal<br>3. Right arm subcutaneous<br>4. Right arm intradermal<br>5. Other (specified)
**Contact detail (encrypted)**	
Full name	(Text)
Date of birth	(Date)
Email / Phone number	(Text)

Supplement Table S2. Survey question – Day 7

Question	Response options
**Introduction**	
This short vaccine safety survey will ask if you had any reactions in the 7 days after your Mpox vaccination.<br><br>This survey is prepared by Australia's national vaccine safety system, AusVaxSafety, and will help us monitor the safety of Mpox vaccines used in Australia. Thank you for your help!	N/A
**Demographics**	
Do you identify as Aboriginal or Torres Strait Islander?	1, Aboriginal<br>2, Torres Strait Islander<br>3, Both<br>0, Neither
What is your gender?	M, Male<br>F, Female<br>O, Another term
Are you currently pregnant?	1. Yes<br>0. No<br>2. Not applicable
**Dose**	
What is the brand of the vaccine?	1. ACAM2000<br>2. Jynneos
Which vaccine dose was this?	1. First<br>2. Second
**Reaction to vaccine**	
Did you have any reaction(s) *in the 7 days after* the vaccination?	1. Yes<br>0. No
Did you need to seek medical care/advice for any of your symptoms?	1. Yes<br>0. No
Please select the type of advice/care that you sought (select all that apply)	1, Phone advice (e.g. HealthDirect)<br>2, Care from a GP, Aboriginal Healthcare Worker or sexual health clinic (in person, telehealth, email, urgent care clinic, home visit)<br>3, Visit to a hospital emergency department

Were you diagnosed with myocarditis and/or pericarditis?	1. Yes<br>0. No<br>9. Unsure
**Reactions**	
Local reaction (pain, redness, swelling, itching at or near the injection site)	1. Yes<br>0. No
Please select all that apply	1, Pain<br>2, Redness<br>3, Swelling<br>4, Itching
Did the swelling extend to/past the elbow or shoulder?	1. Yes<br>0. No
Swelling in armpit area (lymphadenopathy)	1. Yes<br>0. No
Fever	1. Yes<br>0. No
Please indicate the HIGHEST measured temperature (leave blank if not measured)	Free text – numeric
Rash (not at injection site)	1. Yes<br>0. No
Where was the rash? (select all that apply)	1, Face/Head/Neck<br>2, Chest/Back<br>3, Arms<br>4, Legs
Did you have any of the following symptoms at the same time as the rash? (select all that apply)	1, Cough<br>2, Shortness of breath<br>3, Noisy or difficult breathing<br>4, Lip or tongue swelling<br>0, None of these
When did the rash start?	1, Within 1 hour after vaccination<br>2, 1 to 6 hours<br>3, 7 to 72 hours<br>4, More than 72 hours (3 days)
How long did the rash last?	1, Less than 30 minutes

	2, 30 minutes to 24 hours<br>3, More than 24 hours
Chills (shivering and feeling cold)	1. Yes<br>0. No
Headache, muscle/body aches, or joint aches/pain	1. Yes<br>0. No
Please select all that apply	1, Headache<br>2, Muscle/body aches<br>3, Joint aches/pain
Gastrointestinal symptoms (nausea, vomiting, diarrhoea, abdominal pain)?	1. Yes<br>0. No
Please select all that apply	1, Nausea<br>2, Vomiting<br>3, Diarrhoea<br>4, Abdominal pain
Fatigue or tiredness	1. Yes<br>0. No
Fainting/loss of consciousness	1. Yes<br>0. No
Did you have any of the following symptoms at the same time as the faint? (select all that apply)	1, Cough<br>2, Shortness of breath<br>3, Noisy or difficult breathing<br>4, Lip or tongue swelling<br>0, None of these
When did the fainting/loss of consciousness start?	1, Less than 5 minutes after vaccination<br>2, 5 to 30 minutes<br>3, More than 30 minutes
How long did the fainting/loss of consciousness last?	1, Less than 1 minute<br>2, 1 to 5 minutes<br>3, More than 5 minutes
Seizure	1. Yes<br>0. No
When did the seizure start?	1, Less than 30 minutes after vaccination<br>2, Same day

	3, Next day<br>4, 2 or more days after vaccination
How long did the seizure last?	1, Less than 1 minute<br>2, 1 to 5 minutes<br>3, 6 to 15 minutes<br>4, 16 to 30 minutes<br>5, More than 30 minutes
Chest pain, pressure or discomfort?	1. Yes<br>0. No
Shortness of breath	1. Yes<br>0. No
Palpitations	1. Yes<br>0. No
Did you experience any other symptoms not listed above?	1. Yes<br>0. No
Please describe	Free text
**Symptom management**	
Did you take pain or fever medicine (e.g. paracetamol or ibuprofen) *at the time of* vaccination?	1. Yes<br>0. No
Did you use something *after* vaccination to help your symptoms?	1. Yes<br>0. No
Please check all that apply	1, Pain/fever relief (e.g. paracetamol or ibuprofen)<br>2, On skin (e.g. cream, icepack)<br>3, Anti-allergy medicine (e.g. antihistamine)<br>4, Other
If other, please specify	Free text
Do you still have any of the symptoms you reported?	0, No, all of the symptoms have gone<br>1, Yes, one or more symptoms remain
**Health impact**	
Did any of the symptoms you reported cause you to miss work, study, school or normal daily activities?	1. Yes<br>0. No
How many days did you miss?	1, Less than 1 day<br>2, 1 day

	3, 2 days<br>4, 3 or more days
Do you have a history of anaphylaxis or carry an EpiPen?	1. Yes<br>0. No
What was the trigger?	1, Food<br>2, Medicine<br>3, Vaccine<br>4, Insect venom<br>0, Other
If other, please specify	Free text
Do you have any chronic medical conditions?	1. Yes<br>0. No

Please select all that apply	A, Heart disease (coronary heart disease or failure)<br>B, Poorly controlled blood pressure<br>C, Diabetes/sugar<br>D, Chronic lung disease (not including mild/moderate asthma)<br>E, Obesity with BMI > 40 kg/m2<br>F, Chronic kidney failure<br>G, Chronic liver disease<br>H, Cancer (not including blood or bone marrow cancer) diagnosed in the last 12 months<br>I, Blood cancer (e.g. leukaemia, lymphoma or myelodysplastic syndrome) diagnosed within the last 5 years<br>J, Currently receiving chemotherapy or radiotherapy<br>K, Organ transplant recipient on immune suppressive therapy<br>L, Bone marrow transplant recipient in the last 2 years<br>M, Neurological condition (e.g. stroke, dementia)<br>N, Chronic inflammatory conditions (e.g. rheumatoid arthritis, lupus)<br>O, Primary or acquired immunodeficiency (including HIV)<br>P, Other
Please describe	Free text

Supplement Table S3. Survey questions – Day 14

**Question**	**Response options**
**Introduction**	
Thank you for completing the safety survey on reactions after your recent Mpox vaccination.<br><br>This is a short follow-up survey to ask if you had any new or ongoing reactions on days 8 to 14 after your Mpox vaccination.<br><br>If there are symptoms previously reported that have now resolved there is no need to report these again.<br><br>This survey is prepared by Australia's national vaccine safety system, AusVaxSafety, and will help us monitor the safety of Mpox vaccines used in Australia. Thank you for your help!	N/A
**Reaction to vaccine**	
Did you have any reaction(s) *on days 8-14 after* the vaccination?	1. Yes<br><br>0. No
Did any of the new/ongoing symptom(s) cause you to seek advice/care from a doctor/healthcare professional?	1. Yes<br><br>0. No
Please select the type of advice/care that you sought (select all that apply)	1, Phone advice (e.g. HealthDirect)<br><br>2, Care from a GP, Aboriginal Healthcare Worker or sexual health clinic (in person, telehealth, email, urgent care clinic, home visit)<br><br>3, Visit to a hospital emergency department
Were you diagnosed with myocarditis and/or pericarditis?	1. Yes<br><br>0. No<br><br>9. Unsure
**Reactions**	
Local reaction (pain, redness, swelling, itching at or near the injection site)	1. Yes<br><br>0. No
Please select all that apply	1, Pain<br><br>2, Redness<br><br>3, Swelling<br><br>4, Itching

Did the swelling extend to/past the elbow or shoulder?	1. Yes<br>0. No
Swelling in armpit area (lymphadenopathy)	1. Yes<br>0. No
Fever	1. Yes<br>0. No
Please indicate the HIGHEST measured temperature (leave blank if not measured)	Free text – numeric
Rash (not at injection site)	1. Yes<br>0. No
Where was the rash? (select all that apply)	1, Face/Head/Neck<br>2, Chest/Back<br>3, Arms<br>4, Legs
Did you have any of the following symptoms at the same time as the rash? (select all that apply)	1, Cough<br>2, Shortness of breath<br>3, Noisy or difficult breathing<br>4, Lip or tongue swelling<br>0, None of these
When did the rash start?	1, Within 1 hour after vaccination<br>2, 1 to 6 hours<br>3, 7 to 72 hours<br>4, More than 72 hours (3 days)
How long did the rash last?	1, Less than 30 minutes<br>2, 30 minutes to 24 hours<br>3, More than 24 hours
Chills (shivering and feeling cold)	1. Yes<br>0. No
Headache, muscle/body aches, or joint aches/pain	1. Yes<br>0. No
Please select all that apply	1, Headache<br>2, Muscle/body aches<br>3, Joint aches/pain

Gastrointestinal symptoms (nausea, vomiting, diarrhoea, abdominal pain)?	1. Yes<br>0. No
Please select all that apply	1, Nausea<br>2, Vomiting<br>3, Diarrhoea<br>4, Abdominal pain
Fatigue or tiredness	1. Yes<br>0. No
Fainting/loss of consciousness	1. Yes<br>0. No
Did you have any of the following symptoms at the same time as the faint? (select all that apply)	1, Cough<br>2, Shortness of breath<br>3, Noisy or difficult breathing<br>4, Lip or tongue swelling<br>0, None of these
When did the fainting/loss of consciousness start?	1, Less than 5 minutes after vaccination<br>2, 5 to 30 minutes<br>3, More than 30 minutes
How long did the fainting/loss of consciousness last?	1, Less than 1 minute<br>2, 1 to 5 minutes<br>3, More than 5 minutes
Seizure	1. Yes<br>0. No
When did the seizure start?	1, Less than 30 minutes after vaccination<br>2, Same day<br>3, Next day<br>4, 2 or more days after vaccination
How long did the seizure last?	1, Less than 1 minute<br>2, 1 to 5 minutes<br>3, 6 to 15 minutes<br>4, 16 to 30 minutes<br>5, More than 30 minutes
Chest pain, pressure or discomfort?	1. Yes<br>0. No

Shortness of breath	1. Yes<br>0. No
Palpitations	1. Yes<br>0. No
Did you experience any other symptoms not listed above?	1. Yes<br>0. No
Please describe	Free text
**Symptom management**	
Did you take pain or fever medicine (e.g. paracetamol or ibuprofen) *at the time of* vaccination?	1. Yes<br>0. No
Did you use something *after* vaccination to help your symptoms?	1. Yes<br>0. No
Please check all that apply	1, Pain/fever relief (e.g. paracetamol or ibuprofen)<br>2, On skin (e.g. cream, icepack)<br>3, Anti-allergy medicine (e.g. antihistamine)<br>4, Other
If other, please specify	Free text
Do you still have any of the symptoms you reported?	0, No, all of the symptoms have gone<br>1, Yes, one or more symptoms remain
**Health impact**	
Did any of the symptoms you reported cause you to miss work, study, school or normal daily activities?	1. Yes<br>0. No
How many days did you miss?	1, Less than 1 day<br>2, 1 day<br>3, 2 days<br>4, 3 or more days
Do you have a history of anaphylaxis or carry an EpiPen?	1. Yes<br>0. No
What was the trigger?	1, Food<br>2, Medicine<br>3, Vaccine<br>4, Insect venom<br>0, Other

If other, please specify	Free text
Do you have any chronic medical conditions?	1. Yes<br>0. No
Please select all that apply	A, Heart disease (coronary heart disease or failure)<br>B, Poorly controlled blood pressure<br>C, Diabetes/sugar<br>D, Chronic lung disease (not including mild/moderate asthma)<br>E, Obesity with BMI > 40 kg/m2<br>F, Chronic kidney failure<br>G, Chronic liver disease<br>H, Cancer (not including blood or bone marrow cancer) diagnosed in the last 12 months<br>I, Blood cancer (e.g. leukaemia, lymphoma or myelodysplastic syndrome) diagnosed within the last 5 years<br>J, Currently receiving chemotherapy or radiotherapy<br>K, Organ transplant recipient on immune suppressive therapy<br>L, Bone marrow transplant recipient in the last 2 years<br>M, Neurological condition (e.g. stroke, dementia)<br>N, Chronic inflammatory conditions (e.g. rheumatoid arthritis, lupus)<br>O, Primary or acquired immunodeficiency (including HIV)<br>P, Other
Please describe	Free text

Supplement Table S4. Demographic characteristics of study participants (N = 10,943)

Characteristics	MVA-BN 0.1-mL intradermal (N = 6,511)								MVA-BN 0.5-mL subcutaneous given alone (N = 4,432)							
	Non-concomitant (N = 6,401)				Concomitant (N = 110)				Non-concomitant (N = 4,342)				Concomitant (N = 90)			
	Dose 1 (N = 3,675)		Dose 2 (N = 2,726)		Dose 1 (N = 73)		Dose 2 (N = 37)		Dose 1 (N = 2,840)		Dose 2 (N = 1,502)		Dose 1 (N = 61)		Dose 2 (N = 29)	
	n	%	n	%	n	%	n	%	n	%	n	%	n	%	n	%
Age (years)																
18-39	1,735	47.2	1,074	39.4	43	58.9	25	67.6	1,194	42	575	38.3	30	49.2	14	48.3
40-59	1,471	40	1,226	45	26	35.6	9	24.3	1,254	44.2	703	46.8	25	41	6	20.7
≥60	469	12.8	426	15.6	4	5.5	3	8.1	392	13.8	224	14.9	6	9.8	9	31
Gender																
Male	3,571	97.2	2,674	98.1	72	98.6	37	100	2,726	96	1,454	96.8	60	98.4	27	93.1
Female	72	2	37	1.4	1	1.4	0	0	87	3.1	42	2.8	1	1.6	2	6.9
Another term	32	0.9	15	0.6	0	0	0	0	27	1	6	0.4	0	0	0	0
Any chronic medical condition																
Yes	562	15.3	437	16	8	11	6	16.2	512	18	232	15.4	14	23	11	37.9
No	3,113	84.7	2,289	84	65	89	31	83.8	2,328	82	1,270	84.6	47	77	18	62.1

Supplement Table S5. Demographic characteristics of included and excluded participants

Characteristics	Included participants (N = 10,943)		Excluded participants (N = 12,981)			
			Non-respondents to Day 14 survey (N = 4,543)		Non-respondents to Day 7 survey (N = 8,438)	
	n	%	n	%	n	%
Age (years)						
0-17	0	0	2	<0.1	3	<0.1
18-39	4,690	43	2,613	58	5,290	63
40-59	4,720	43	1,606	35	2,716	32
≥60	1,533	14	322	7.1	429	5.1
Gender						
Male	10,621	97	4,367	96	8,071	96
Female	242	2.2	126	2.8	211	2.5
Another term	80	0.7	50	1.1	156	1.9
Any chronic medical condition						
Yes	1,782	16	645	14	-	-
No	9,161	84	3,898	86	-	-
Vaccine route						
Intradermal	6,511	59	2,813	62	4106	49
Subcutaneous	4,432	41	1,728	38	4332	51
Unknown	0	0	2	<0.1	0	0
Vaccine dose						
Dose 1	6,649	61	3,162	70	4,398	51
Dose 2	4,294	39	1,381	30	4,119	49
Unknown	0	0	0	0	11	0.1
Concomitant vaccination status						
Non-concomitant	10,743	98	4,454	98	-	-
Concomitant	200	1.8	89	2.0	-	-
